# Clinicopathological findings, prognosis, and Epstein–Barr virus infection in rheumatoid arthritis patients with other iatrogenic immunodeficiency-associated T- and NK-cell lymphoproliferative disorders

**DOI:** 10.1186/s12885-022-10358-0

**Published:** 2022-12-22

**Authors:** Shoichi Kimura, Yumi Oshiro, Hiromi Iwasaki, Masanori Kadowaki, Masao Ogata, Tsutomu Daa, Toshifumi Sakata, Shigeto Kawauchi, Ziyao Wang, Yasushi Takamatsu, Morishige Takeshita

**Affiliations:** 1 Graduate School of Medical Sciences, Division of Pathomorphology, Fukuoka University, 7-45-1 Nanakuma, Jonan-ku, Fukuoka, 8140180 Japan; 2 Department of Pathology, Faculty of Medicine, and Fukuoka University, 7-45-1 Nanakuma, Jonan-ku, Fukuoka, 8140180 Japan; 3 Department of Pathology, Matsuyama Red Cross Hospital, 1 Bunkyo-cho, Matsuyama, 7910000 Japan; 4 Department of Haematology, Clinical Research Centre, National Hospital Organization Kyushu Medical Centre, 1-8-1 Jigyohama, Chuo-ku, Fukuoka, 8108563 Japan; 5 Department of Hematology, Faculty of Medicine, Oita University, Idaigaoka, Hazama-machi, Yufushi, Oita, 8795593 Japan; 6 Department of Pathology, Faculty of Medicine, Oita University, Idaigaoka, Hazama-machi, Yufushi, Oita, 8795593 Japan; 7 Department of Otolaryngology, Faculty of Medicine, and Fukuoka University, 7-45-1 Nanakuma, Jonan-ku, Fukuoka, 8140180 Japan; 8 Department of Pathology, Clinical Research Centre, National Hospital Organization Kyushu Medical Centre, 1-8-1 Jigyohama, Chuo-ku, Fukuoka, 8108563 Japan; 9 Department of Internal Medicine, Division of Medical Oncology, Hematology and Infectious Disease, Faculty of Medicine, Fukuoka University, 7-45-1 Nanakuma, Jonan-ku, Fukuoka, 8140180 Japan

**Keywords:** Rheumatoid arthritis, Iatrogenic immunodeficiency, Methotrexate, T cells, NK cells, lymphoproliferative disorders, Epstein–Barr virus

## Abstract

**Background:**

Other iatrogenic immunodeficiency-associated (OIIA) T- and natural killer (NK)-cell lymphoproliferative disorders (TNK-LPDs) are rare in patients with rheumatoid arthritis (RA).

**Methods:**

We investigated the clinicopathological characteristics, Epstein–Barr virus (EBV) infection, genetic findings, therapeutic response, and prognostic factors in 21 RA patients with OIIA TNK-LPDs and compared these with those of 39 with OIIA B-cell LPDs (B-LPDs) and 22 with non-OIIA B-LPDs.

**Results:**

Immunohistologically, 11 patients (52%) showed CD4+ T-LPDs, and 7 had a T follicular helper (TFH) phenotype. The other nine patients (43%) showed CD8+ T-LPDs, and the remaining one (5%) had features of CD3+ CD4− CD8− nasal type TNK-cell lymphoma. CD30+, p53+, and CMYC+ atypical lymphocytes were identified in seven (33%), eight (38%), and five (24%) patients, respectively. In situ hybridisation detected EBV-encoded RNA (EBER) + large atypical lymphocytes in five patients (24%). Nine of 17 patients (53%) showed clonal peaks of *TCRγ* by polymerase chain reaction. Withdrawal of MTX and biologic drugs was effective in 12 patients (57%), and 8 (38%) received chemotherapies. Two patients with TFH+ or EBV+ CD4+ CD30+ large cell peripheral T-cell lymphoma, one with CD8+ systemic anaplastic large cell lymphoma, and two with systemic EBV+ CD8+ T-cell lymphoma of childhood showed a lethal progressive clinical course within 13 months. Moreover, > 500 U/L LDH, large atypical lymphocytes, expression of CD30, p53, and CMYC, and EBER+ atypical lymphocytes were significantly poor prognostic factors for overall survival (*p* < 0.05). Median interval from RA onset to OIIA TNK-LPDs was 72 months, which was shorter than 166 months in OIIA B-LPDs (*p* = 0.003). EBV+ atypical and reactive lymphocytes were frequently found in 15 patients with OIIA TNK-LPDs (71%), in 27 with OIIA B-LPDs (69%), and only in 3 with non-OIIA B-LPDs (14%).

**Conclusions:**

OIIA TNK-LPDs occurred in early phase of RA, compared with OIIA B-LPDs, and occasionally showed a lethal progressive clinical course. Detection of OIIA TNK-LPD patients with poor prognostic factors is necessary. EBV infection in immunosuppressed patients due to persistent RA, MTX, and biologic drugs may play a role in forming the tumour microenvironment and lymphomagenesis of TNK-LPDs.

**Supplementary Information:**

The online version contains supplementary material available at 10.1186/s12885-022-10358-0.

## Background

Post-transplant lymphoproliferative disorders (LPDs) are found in recipients with immunosuppressants, and other iatrogenic immunodeficiency-associated (OIIA) LPDs mainly occur in rheumatoid arthritis (RA) patients treated with low-dose methotrexate (MTX), tumour necrosis factor (TNF) inhibitors, and other biologic drugs, and are infrequent in other types of autoimmune disease [1]. The histological types of OIIA LPDs in RA patients are mainly composed of B-cell LPDs (B-LPDs), occasionally Hodgkin lymphoma, and rarely T- and natural killer (NK)-cell LPDs (TNK-LPDs) [1–4]. OIIA TNK-LPDs are mostly composed of CD8+ T-large granular lymphocytosis/leukaemia (T-LGL), angioimmunoblastic T-cell lymphoma (AITL) with CD4+ T follicular helper (TFH) phenotype, peripheral T-cell lymphoma (PTCL)-not otherwise specified (NOS), or T-LPDs, and rarely of nasal type TNK-cell lymphoma (TNKCL) [5–9]. Approximately 4% of RA patients treated with or without MTX, TNF inhibitors, and other biologic drugs showed clonal expansions of mainly CD8+ and occasionally CD4+ T-LGL [10]. Epstein–Barr virus-encoded RNA (EBER) + atypical lymphocytes are frequently observed in OIIA B-LPDs and Hodgkin lymphoma, but are rarely reported in TNK-LPDs and T-LGL [1, 3, 4, 7, 8]. Furthermore, EBV+ atypical lymphocytes indicated a favourable prognostic factor in patients with OIIA B-LPDs but were not evident in TNK-LPDs [3, 4, 8]. Withdrawal of MTX, TNF inhibitors, and other biologic drugs are effective for the treatment of RA patients with OIIA B- and TNK-LPDs, and additional chemotherapies are also effective in patients without tumour regression [4, 8]. However, a lethal T-cell lymphoma (TCL) has been reported in a few RA patients treated with MTX and biologic drugs [7, 11, 12]. Three RA patients treated with MTX and TNF inhibitors and 22 with inflammatory bowel disease (IBD) receiving TNF inhibitors had hepatosplenic TCL with a lethal progressive clinical course, as recorded in the database of Food and Drug Administration (FDA) adverse event reporting system [13]. MTX, TNF inhibitors, and other biologic drugs may induce profound immunosuppression and possibly lethal lymphomagenesis in RA patients.

In the current study, we subclassified 21 RA patients with OIIA TNK-LPDs into CD4+, CD8+, and CD4− CD8− phenotypes, and identified characteristic histological and immunohistological findings to EBV infection of atypical lymphocytes and scattered lymphocytes in the background, genetic findings, and prognostic factors, which were compared with those of OIIA and non-OIIA B-LPDs. We advocated detection of TNK-LPDs with poor prognostic factors including EBER+ atypical lymphocytes, and examined the etiological role of EBV infection in RA patients with OIIA TNK-LPDs.

## Methods

### Patient selection and clinical findings

Twelve thousand three hundred registered patients with LPDs or lymphomas were reviewed and retrieved retrospectively over the period of 1990 to 2020 at the Department of Pathology, Fukuoka University. Histological classification was performed according to the World Health Organization classification (2017) [1]. We retrospectively reviewed the medical records of the patients who met all the inclusion criteria. The inclusion criteria included patients who met the American College of Rheumatology classification criteria for RA; we selected RA patients who developed LPDs. OIIA LPDs were defined as LPDs occurring in RA patients treated with low-dose MTX, TNF inhibitors, or other biologic drugs [1, 9]. Twenty-one RA patients with OIIA TNK-LPDs, 39 RA patients with OIIA B-LPDs, and 22 RA with non-OIIA B-LPDs were analysed. Human immunodeficiency virus (HIV)-positive patients and human T-lymphotropic virus (HTLV)-1-positive carriers were not included in the current study. Corresponding medical records were reviewed to obtain clinical information, including Ann Arbor stage, treatment, and survival. The criteria of LPD regression were if LPDs in RA patients had shrunk or improved following 4-week withdrawal of MTX, biologic drugs, and immunosuppressants, according to the systemic findings including laboratory data and radiological images, and did not require further treatment with chemotherapy [14]. Diagnosis of hemophagocytic lymphohistiocytosis (HLH) was made using the revised HLH-2004 criteria [15].

### Histology, immunohistology, and detection of EBV

Excised tissue specimens were fixed in 10% formalin to generate formalin-fixed paraffin-embedded (FFPE) samples that were stained with haematoxylin and eosin. For immunohistochemistry, antibodies were applied to the tumour samples using a Leica Bond III-automated stainer (Leica Biosystems, Buffalo Grove, IL, USA), and peroxidase reactions were developed using diaminobenzidine. These antibodies are listed in Table S1. Criteria of small, medium, and large atypical cell sizes were compliant with those of mantle cells, centrocytes, and centroblasts, respectively, in lymphoid follicles. Large-cell TNK-LPDs were characterised by ≥50% large lymphoid cells with distinct nucleoli. Small-cell TNK-LPDs consisted of predominantly small- or medium-sized atypical lymphocytes. For all immunostaining, reactions were considered positive when ≥30% of atypical lymphocytes were positively stained. The TFH phenotype was defined by the expression of at least two of the following five antibodies: PD-1, inducible T-cell co-stimulator (ICOS), BCL6, C-X-C motif chemokine ligand (CXCL)13, and CD10 [16]. Assessments of CMYC+, p53+, or programmed cell death-ligand (PD-L) 1+ atypical lymphocytes and the number of PD-L1+ non-neoplastic cells were performed according to the methods reported in our previous study [17]. The presence of EBV infection was determined by in situ hybridisation of EBER+ nuclear signals in ≥50% of atypical lymphoid cells. For EBER staining, tissue sections were hybridised in a solution of 50% formamide containing fluorescein isothiocyanate-labelled EBER oligonucleotides (Leica). Double-staining of lymphocyte markers and EBER was performed to confirm the presence of EBV+ T or B cells.

### Molecular analysis of the *T-cell receptor* (*TCR) γ* locus

For polymerase chain reaction (PCR) amplification of the *TCR*γ locus, DNA was prepared from the FFPE specimens of 17 patients. BIOMED-2 multiplex PCR assays (InVivoScribe Technologies, San Diego, CA, USA) of *TCR*γ (Bottles A, B; Vγ and Jγ gene rearrangements) were performed using standardised protocols and primers [18]. Following amplification, *TCR*γ PCR products were analysed using GeneScan (MultiNA; Shimazu Co., Kyoto, Japan) as previously described [17].

### Detection of *RHOA* G17V mutation by Sanger sequencing

PCR was performed with AmpliTaq gold (Thermo Fisher Scientific, Waltham, MA, USA) using 40 ng genomic DNA, 0.3 μM primers, and 2 μL AmpliTaq gold master mix. A PCR-amplified product of 244 bp, including the codon for the 17th amino acid, was obtained in 12 TLPD patients, and direct sequencing of these products was performed. The coding DNA position 50G > T mutation of the *RHOA* gene predicted the change of the wild-type G (Gly) to the mutant type V (Val) [19].

### Statistical analysis

The clinicopathological features of patients with OIIA TNK-LPDs, OIIA B-LPDs, and non-OIIA B-LPDs were compared with Fisher’s exact test or χ^2^ test. Medians were compared with Wilcoxon rank sum test. For the RA patients with LPDs, progression-free survival (PFS) was calculated from the initial diagnosis date to the first date of disease progression or relapse, and overall survival (OS) was calculated from the initial time of diagnosis to the date of last follow-up or death. PFS and OS curves were generated using the Kaplan–Meier method and analysed by the proportional hazards model. A *p*-value of < 0.05 was considered statistically significant. Statistical analyses were performed using JMP 10 software (SAS Institute, Cary, NC, USA).

## Results

### Clinical findings

The clinical features and treatments of 11 RA patients with OIIA CD4+ T-LPDs (52%), 9 with CD8+ T-LPDs (43%), and 1 with CD4− CD8− TNK-LPDs (5%) are shown in Tables 1 and 2. The median age of all 21 patients was 64 years, and the male to female ratio was 6 to 15. The median interval from RA onset to TNK-LPDs was 72 months, and the median duration of low-dose MTX therapy was 53 months. Overall, ≥ 300 U/L lactate dehydrogenase (LDH) and ≥ 2000 U/ml of soluble interleukin 2 receptor (sIL2R) titres in sera were detected in 13 TNK-LPD patients (62%) and 11 of the examined 19 (58%), respectively. Extranodal tumour involvement was initially identified in six of nine patients with CD8+ T-LPDs (67%), compared with one with CD4+ T-LPDs (9%) (*p* = 0.017). Four patients with CD8+ T-LPDs had bone marrow invasion by atypical lymphocytes, and each one had hepatic (Fig. 1) and subcutaneous tumours. In addition to MTX therapy for RA, one patient with TFH+ PTCL (Case No. 3) and another with CD4+ large cell PTCL-NOS (Case No. 8) received TNF inhibitor (adalimumab) or anti-IL6 receptor antibody (Ab) (tocilizumab) and azathioprine. Two patients with CD8+ HLH and subcutaneous panniculitis-like T-LPDs (Case Nos. 18 and 20) received TNF inhibitor (infliximab) or anti-cytotoxic T lymphocyte antigen (CTLA)-4 Ab (abatacept). Sixteen TNK-LPD patients (76%) were classified with advanced clinical stage III or IV.

**Table 1:** Clinical features, therapies, and prognosis of 21 rheumatoid arthritis (RA) patients with other iatrogenic immunodeficiency-associated (OIIA) T- and NK-cell lymphoproliferative disorders (LPDs)

No.	Age, onset of LPDs (y)	Sex	RA duration (months)	MTX duration (months)	Other biologic drugs for RA	LDH (U/L)	sIL2R (U/ml)	Clinical stage	Regression of LPDs^a^	Chemotherapies	Follow-up (months)/ outcome
1	83	M	78	60	-	520	1316	III	−	VP-16, THP-COP	13/d
2	77	F	48	48	-	208	604	II	−	THP-COP, Rad	63/d
3	71	F	37	36	TNF inhibitor (adalimumab)	130	1480	III	−	CHOP	66/a
4	54	F	36	36	-	196	559	IV	−	CHOP	23/a
5	66	M	11	11	-	167	464	III	+	−	102/a
6	77	F	80	70	-	296	402	II	+	−	55/a
7	63	F	168	168	-	162	429	IV	+	−	70/a
8	47	M	48	48	Anti-IL6 receptor Ab (tocilizumab)	567	18029	IV	−	Brentuximab vedotin	8/d
9	68	F	70	70	-	272	nt	II	+	−	31/a
10	70	M	80	4	-	547	8010	IV	+	−	58/d
11	73	F	12	12	-	657	11165	III	+	−	117/a
12	69	F	84	36	-	598	58090	IV	−	THP-COP	2/d
13	77	F	168	108	-	1590	2662	IV	−	-	1/d
14	59	F	36	36	-	1915	21816	IV	−	THP-COP	4/d
15	61	F	150	57	-	503	8088	IV	+	−	156/a
16	45	F	31	31	-	370	817	IV	+	−	107/a
17	58	M	29	29	-	521	4523	I	+	−	3/a
18	59	F	52	39	TNF inhibitor (infliximab)	1794	nt	IV	−	VP-16, CSA	102/a
19	81	F	25	25	-	334	2291	III	+	−	13/a
20	31	M	70	60	Anti-CTLA4 Ab (abatacept)	700	2010	I	+	−	129/a
21	55	F	204	120	-	216	1410	III	+	−	157/a
Total	64 (median)	M/F: 6/15	72 (median)	53 (median)	na	584 (median)	7588 (median)	I/II/III/IV: 2/3/6/10	12	8	na

*Ab* antibody, *CTLA* cytotoxic T lymphocyte antigen, *IL* interleukin, *TNF* tumour necrosis factor, *LDH* lactate dehydrogenase, *MTX* methotrexate, *sIL-2R* soluble interleukin-2 receptor, *CHOP* cyclophosphamide, doxorubicin, vincristine, prednisone, *CSA* cyclosporine A, *Rad* radiation, *THP-COP* pirarubicin, cyclophosphamide, vincristine, prednisolone, *VP-16* etoposide

^a^regression of LPDs by withdrawal of MTX and biologic drugs. a, alive; d, dead, na, not available

**Table 2:** Histological, immunohistological findings and *TCRγ* gene in 21 rheumatoid arthritis (RA) patients with other iatrogenic immunodeficiency-associated (OIIA) T- and NK-cell lymphoproliferative disorders (LPDs)

No.	Biopsy site	Atypical lymphoid cell size	Phenotype of atypical lymphocytes	TFH markers (#)	CD30	p53	CMYC	EBV (Latency)	TCR βF1	*TCRγ* gene clonality	Histological diagnosis
1	LN	Large	CD4+	+ (3)	−	+	+	−	+	+	TFH+ PTCL
2	Pharynx	Large	CD4+	+ (4)	+	+	−	−*	−	+	TFH+ PTCL
3	LN	Large	CD4+	+ (5)	+	+	−	−**	+	−	TFH+ PTCL
4	LN	Small	CD4+	+ (4)	−	−	−	−*	−	+	TFH+ PTCL
5	LN	Small	CD4+	+ (2)	−	−	−	−*	+	−	TFH+ T-LPDs
6	LN	Small	CD4+	+ (2)	−	−	−	−**	+	−	TFH+ T-LPDs
7	LN	Small	CD4+	+ (2)	−	−	−	−*	+	−	TFH+ T-LPDs
8	LN	Large	CD4+, CD3-	− (0)	+	+	+	+ (II)	−	+	PTCL-NOS
9	LN	Large	CD4+	− (0)	+	−	−	+ (II)	−	nt	PTCL-NOS
10	LN	Small	CD4+	− (1)	−	−	−	−	+	−	T-LPDs
11	LN	Small	CD4+	− (1)	−	−	−	−*	+	−	T-LPDs
12	LN	Large	CD8+	− (0)	+	+	+	−*	+	nt	systemic ALCL
13	Liver	Large	CD8+	− (1)	+	+	−	+ (II)	−	nt	systemic EBV+ TCL of childhood
14	BM	Large	CD8+	− (1)	−	+	+	+ (I)	+	+	systemic EBV+ TCL of childhood
15	BM, LN	Small	CD8+	− (1)	ー	−	−	−	+	+	T-LGL
16	BM	Small	CD8+	− (0)	−	−	−	−	+	+	T-LGL
17	LN	Small	CD8+	− (0)	−	−	−	−**	+	+	PTCL-NOS
18	BM	Small	CD8+	− (1)	−	−	−	−	−	nt	HLH
19	LN	Small	CD8+	− (1)	−	−	−	−*	+	−	T-LPDs
20	Subcutis	Small	CD8+	− (1)	−	−	−	−	+	−	Panniculitis-like T-LPDs
21	Nose	Large	CD4−, CD8−	− (1)	+	+	+	+ (II)	+	+	Nasal type TCL
Total	na	na	na	7	7	8	5	5	15	9/17	na

*ALCL* anaplastic large cell lymphoma, *BM* bone marrow, *EBV* Epstein–Barr virus, *HLH* hemophagocytic lymphohistiocytosis, *LGL* large granular lymphocytosis, *LN* lymph node, *NOS* not otherwise specified, *PTCL* peripheral T-cell lymphoma, *TCL* T-cell lymphoma, *TCR* T-cell receptor, *TFH* T follicular helper

#, positive markers in examined five antibodies; +, tumour cell-positive; *, **, scattered EBER+ small (*) and large (**) lymphocytes in background; na, not available, nt, not test.

**Fig. 1:**
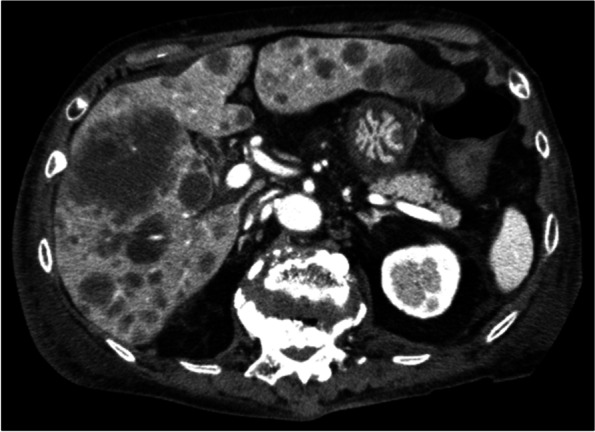
Multiple hepatic nodules were detected in a patient with systemic EBV+ CD8+ TCL (Case No. 13) by computed tomography. The maximum nodule size was 78 mm, and no lymphadenopathy was found in the abdomen

### Histology, immunohistological findings, EBV, and genetic studies

**Fig. 2:**
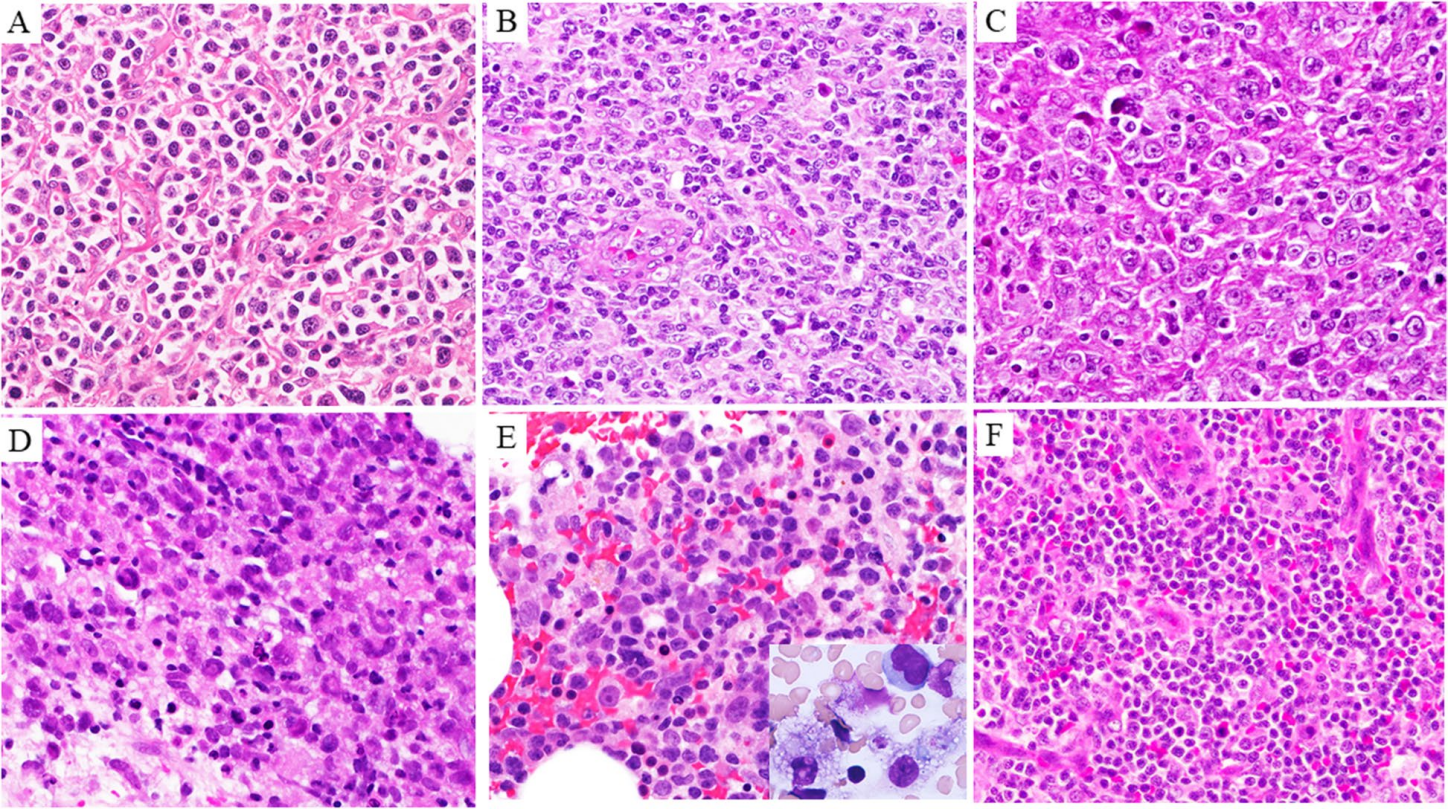
**A**: Diffuse invasion by large, atypical lymphocytes in nodal CD4+ TFH+ PTCL (Case No. 1). **B**: Diffuse infiltrates of small, atypical lymphocytes and plasma cells and moderate vascular reactions were found in nodal CD4+ TFH+ T-LPDs (Case No. 6). **C**: Diffuse infiltrate of large, atypical lymphocytes with round nuclei and distinct nucleoli in nodal EBER+ CD4+ PTCL-NOS (Case No. 8). Intrahepatic invasion by large, atypical lymphocytes with lobulated nuclei were observed (**D**), along with bone marrow invasion by medium-sized and large, atypical lymphocytes and two phagocytic macrophages (**E**) in systemic EBV+ CD8+ TCL (Case Nos. 13 and 14). **F**: Diffuse monotonous infiltrate of small lymphocytes with mild atypia in nodal CD8+ PTCL-NOS (Case No. 17). HE staining, × 400

**Fig. 3:**
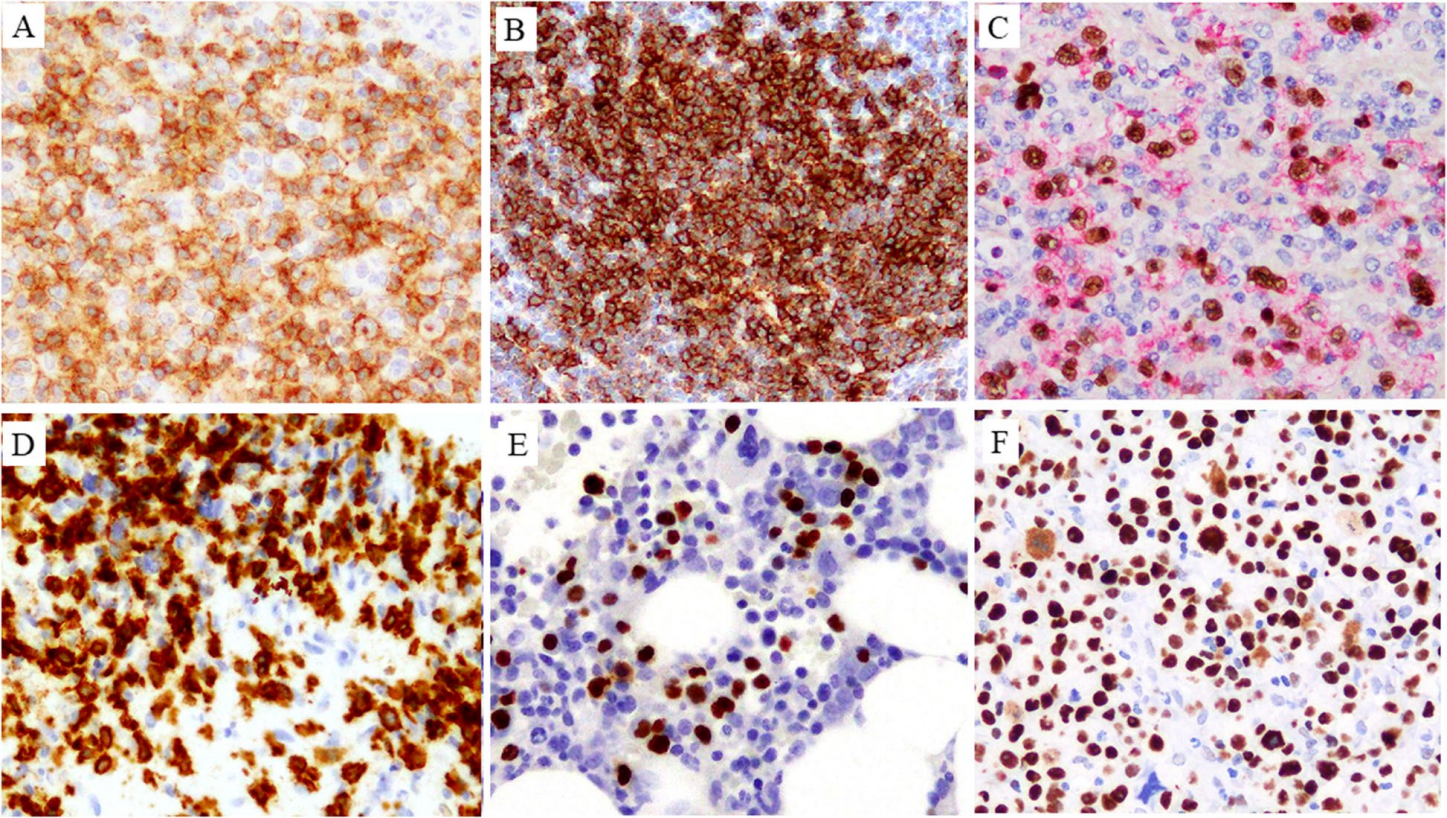
CD4+ (**A**) and ICOS+ (**B**) large- or medium-sized lymphocytes were observed in nodal TFH+ PTCL (Case Nos. 3 and 4). **C**: Many CD30+ (red) and EBER+ (brown) large, atypical lymphocytes were detected in PTCL-NOS (Case No. 8). **D**: CD8+ large lymphocytes were diffusely found in liver of sEBV+ CD8+ TCL (Case No. 13). **E**: EBER+ medium- to large-sized lymphocytes were partly clustered in the bone marrow of sEBV+ CD8+ TCL (Case No 14). **F**: Many p53+ large, atypical lymphocytes were detected in nodal CD8+ sALCL (Case No. 12)

**Fig. 4:**
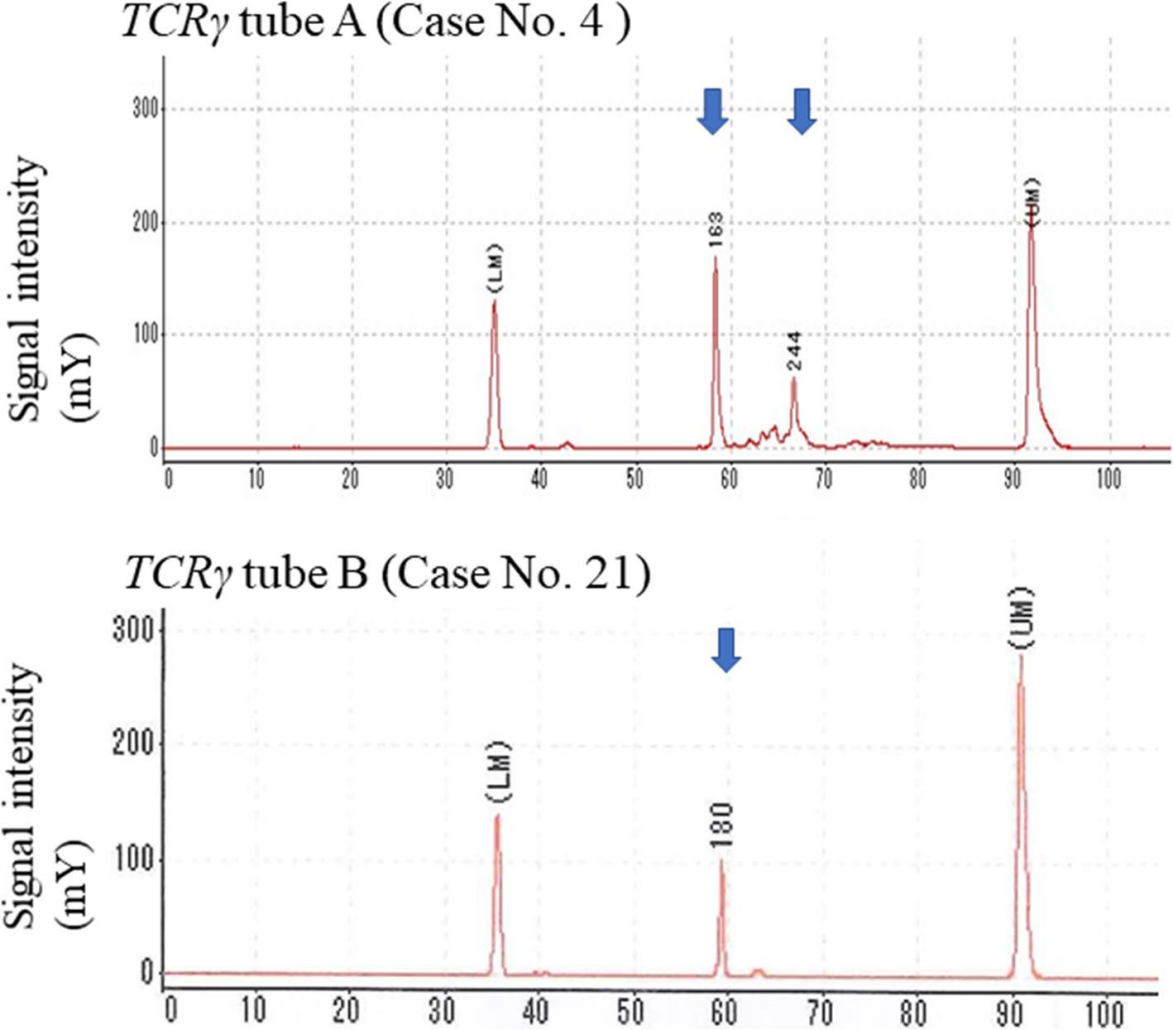
T-cell receptor *(TCR)* γ gene analysis by PCR. Clonal peaks (arrows) of the *TCR*γ gene Vγ and Jγ regions (Tubes A, B) detected by GeneScan analysis in Case Nos. 4 and 21, respectively. The lower marker (LM) and upper marker (UM) were spiked as internal standards

Histologically, the tumours of 9 patients (43%) mainly consisted of large, atypical lymphocytes, and the remaining 12 (57%) were composed of small- and/or medium-sized atypical lymphocytes, as shown in Table 2. In 11 CD4+ T-LPD patients, 7 (Case Nos. 1–7) showed diffuse invasion by CD4+ large or small, atypical lymphocytes with the TFH phenotype (Fig. 2A, 3A). Moderate plasmacytic and histiocytic reactions, vascular reaction, and partly preserved CD20+ B cell nests were found in four patients with small cell TFH+ T-LPDs (Case Nos. 4–7), and no definite nests of neoplastic clear cells were detected (Fig. 2B). Among TFH+ T-LPDs, atypical lymphocytes showed ICOS expression in seven patients (100%, Fig. 3B), PD-1 in six (86%), CXCL13 in four (57%), BCL6 in three (43%), and CD10 in two (29%). The other two patients (Case Nos. 8 and 9) had CD4+ EBER+ large-cell PTCL-NOS with expression of CD30 and CD25 and without the TFH phenotype (Figs. 2C, 3C). The remaining two patients (Case Nos. 10 and 11) showed CD4+ small-cell T-LPDs without the TFH phenotype. In nine patients with CD8+ T-LPDs, one (Case No. 12) had CD3+ EBER−sALCL with expression of CD30, CD25, and TIA1. Two patients (Case Nos. 13 and 14) had CD8+ T-LPDs mainly involving the bone marrow and liver, revealing features of systemic EBV (sEBV) + TCL of childhood with HLH (Fig. 2D, E, 3D, E). The remaining six patients (Case Nos. 15–20) had CD8+ and TIA1+ T-LPDs (Fig. 2F), manifesting T-LGL, bone marrow-involved HLH, nodal small-cell TCL or T-LPDs, and subcutaneous panniculitis-like T-LPDs. The remaining patient (Case No. 21) had CD3+ CD4− CD8− CD56− EBER+ nasal type TNKCL with CD30, CD25, and TIA1 expression in the nasal cavity. CD30+ large, atypical lymphocytes were detected in six CD4+ or CD8+ TCL patients and in one nasal type TNKCL (5%), and the atypical lymphocytes were negative for anaplastic lymphoma kinase (ALK). p53+ atypical lymphocytes were detected in eight patients (38%) (Fig. 3F), and CMYC+ atypical lymphocytes were found in five (24%). PD-L1+ atypical lymphocytes were detected in three patients (14%); one each of CD4+ large-cell PTCL-NOS, sEBV+ CD8+ TCL, and CD4− CD8− nasal type TNKCL. Intense (R3+) reactions of PD-L1+ non-neoplastic cells were detected in the other four T-LPD patients (19%). Many EBER+ CD20− ENBA2− large, atypical lymphocytes were detected in five patients (24%), and four of them were positive for CD30, CD3, and LMP1. Latency type II EBV infection was found in four patients, and the remaining one (Case No. 14) showed latency type I. Scattered EBER+ small CD20+ lymphocytes were detected in the other 10 patients with CD4+ and CD8+ T-LPDs (48%). Among them, some EBER+ CD20+ large lymphocytes were identified in two CD4+ TFH+ patients and one CD8+ T-LPD patient. Fifteen patients (71%) showed TCRβF1+ atypical lymphocytes. Clonal peaks of *TCRγ* gene V*γ* and Jγ regions were detected by PCR in 4 of 10 CD4+ patients (40%), 4 of 6 CD8+ patients (67%), and 1 CD4− CD8− TNK-LPD patient (Fig. 4). *RHOA* p.G17V mutation by Sanger sequencing was detected in only one TFH+ PTCL patient (Case No. 4) among seven TFH+ T-LPDs (14%), but not in two CD4+ PTCL-NOS and three CD8+ T-LPD patients.

### Treatment, prognosis, and prognostic factors

**Fig. 5:**
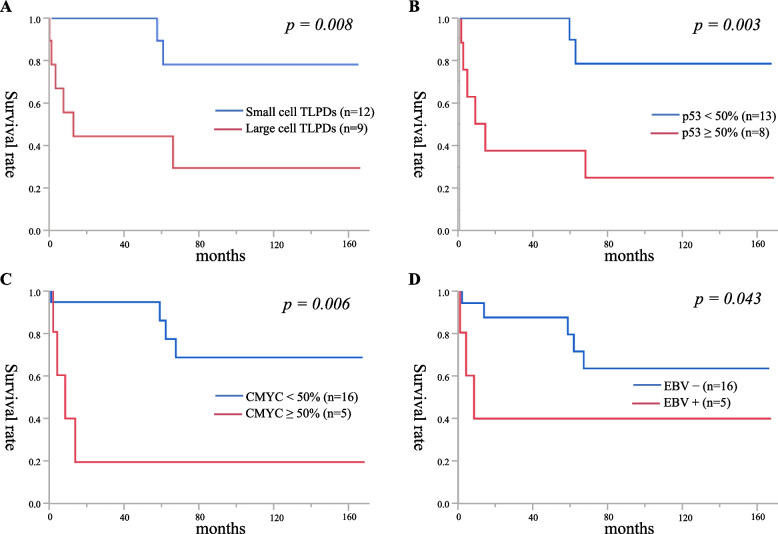
**A**: Nine patients with large-cell TNK-LPDs showed significantly worse OS than 12 with small-cell TNK-LPDs (*p* = 0.008). **B**: Eight patients with p53+ atypical lymphocytes showed significantly worse OS than 13 with p53− atypical lymphocytes (*p* = 0.003). **C**: Five patients with CMYC+ atypical lymphocytes showed significantly worse OS than 16 with CMYC− atypical lymphocytes (*p* = 0.006). **D**: Five patients with EBER+ atypical lymphocytes showed a significantly poorer OS than 16 with EBER− atypical lymphocytes (*p* = 0.043)

Regression of LPDs by withdrawal of MTX and biologic drugs was detected in 12 patients (57%), including 6 CD4+ T-LPDs, 5 CD8+, and 1 CD4− CD8− TNK-LPD, as shown in Table 1. Chemotherapy including cyclophosphamide, doxorubicin, vincristine, and prednisone (CHOP) or pirarubicin, cyclophosphamide, vincristine, and prednisolone (THP-COP), Brentuximab vedotin and etoposide (VP16) were administered to eight patients (38%); among them, five CD4+ T-LPDs and three CD8+ were included. The lethal patient with sEBV+ CD8+ TCL (Case No 13) received only corticosteroid therapy due to their poor clinical condition. Recurrence of T-LPDs was detected in three CD4+ T-LPD patients (14%) (Case Nos 4, 6, 10) and one CD8+ HLH (5%) (Case No 18). Seven patients (33%) died of disease. Among them, two patients with EBV− CD4+ TFH+ PTCL and EBV+ CD4+ CD30+ large-cell PTCL-NOS, one EBV− CD8+ sALCL, and two sEBV+ CD8+ TCLs showed a lethal progressive clinical course within 13 months. Table 3 presents the univariate analysis of prognostic factors in the PFS and OS of 21 TNK-LPD patients. In PFS, ≥ 500 U/L LDH (*p* = 0.038), large, atypical lymphocytes (*p* = 0.01), p53+ (*p* = 0.003), and CMYC+ (*p* = 0.009) atypical lymphocytes indicated significantly worse prognosis. Five-year OS of 11 CD4+ and 9 CD8+ T-LPD patients was 47 and 65%, respectively. In OS, ≥ 500 U/L LDH (*p* = 0.037), large, atypical lymphocytes (*p* = 0.008), CD30+ (*p* = 0.034), p53+ (*p* = 0.003), CMYC+ (*p* = 0.006), and EBER+ (*p* = 0.043) atypical lymphocytes indicated significantly worse prognosis (Fig. 5).

**Table 3:** Univariate analysis of prognostic factors for progression-free and overall survival in 21 rheumatoid arthritis patients with other iatrogenic immunodeficiency-associated (OIIA) T- and NK-cell lymphoproliferative disorders

Risk factors	Univariate analysis of		Univariate analysis of	
	progression free survival		overall survival	
	Hazard ratio	*p*-value	Hazard ratio	*p*-value
Age ≥ 70 years	2.771	0.301	2.225	0.481
LDH ≥ 500 U/L	3.919	0.038	3.849	0.037
sIL2R ≥ 2000 U/ml	2.367	0.135	3.497	0.079
Stages III and IV	1.309	0.474	2.474	0.25
Large atypical lymphocytes	5.808	0.01	6.052	0.008
CD30 ≥ 30%	3.173	0.054	3.982	0.034
TFH phenotype	0.964	0.685	0.642	0.423
p53 ≥ 50%	7.114	0.003	7.007	0.003
CMYC ≥ 50%	5.487	0.009	5.682	0.006
PD-L1 n+	3.022	0.076	3.501	0.057
PD-L1 n+ and R3+	2.087	0.257	1.395	0.519
EBER+ atypical lymphocytes ≥ 50%	3.173	0.054	3.439	0.043
*TCRγ* gene clonality	2.629	0.175	4.962	0.086

*EBER* EBV-encoded RNA, *LDH* lactate dehydrogenase, *n+* tumour cell-positive, *PD-L1* programmed cell death-ligand, *R* reaction, *sIL2R* soluble interleukin 2 receptor, *TCR* T cell receptor, *TFH* T follicular helper

*p-*values were analysed using the generalized Wilcoxon method

### Clinicopathological differences between RA patients with OIIA TNK-, OIIA-, and non-OIIA B-LPDs

As shown in Table 4, the median interval from RA onset to OIIA TNK-LPDs was 72 months, which was significantly shorter than 166 months in OIIA B-LPDs (*p* = 0.003) and 177 months in non-OIIA B-LPDs (*p* = 0.0007). Ten of 19 TNK-LPD patients (53%) showed ≥2000 U/ml of sIL2R, which was significantly higher than six of 28 B-LPDs (21%) (*p* = 0.025). Sixteen patients with OIIA TNK-LPDs showed advanced clinical stages III and IV, which was significantly higher than 17 of OIIA B-LPDs (44%) (*p* = 0.032). As well as MTX treatment, TNF inhibitors were administered to five RA patients with OIIA B-LPDs. Infliximab and anti-IL6 receptor Ab (tocilizumab), anti-IL17 Ab (secukinumab), or anti-CTLA-4 Ab (abatacept) were used in each patient. Azathioprine and cyclosporine were also administered to the other three patients. Five TNK-LPD patients (24%) had ≥50% EBER+ atypical lymphocytes, which was significantly lower than 21 of 39 OIIA B-LPDs (54%) (*p* = 0.031). However, EBER+ atypical lymphocytes and scattered EBER+ small lymphocytes were frequently found in 15 patients with TNK-LPDs (71%) as well as in 27 with OIIA B-LPDs (69%), respectively. Eight patients with TNK-LPDs (38%) received chemotherapy, and 20 with OIIA B-LPDs (51%) received CHOP with rituximab (17 patients) and CHOP or THP-COP (3 patients). Patients with TNK- and OIIA B-LPDs had no prognostic difference in OS (*p* = 0.083). Five patients with EBER+ TNK-LPDs showed significantly worse prognosis than 21 with EBER+ OIIA B-LPDs (*p* = 0.0001). EBV+ atypical lymphocytes was a significantly favourable prognostic factor in 39 patients with OIIA B-LPDs (*p* = 0.015). In 22 RA patients with non-OIIA B-LPDs, EBER+ atypical lymphocytes or scattered EBER+ lymphocytes were noted in 3 patients (14%), which was significantly lower than in those with TNK-LPDs (*p* = 0.0002*)*. Twenty patients with non-OIIA B-LPDs (91%) received chemotherapies mainly of CHOP with rituximab (10 patients) and CHOP (6 patients), and the 5-year OS was 40%. Patients with TNK-LPDs and non-OIIA B-LPDs had no prognostic difference in OS (*p* = 0.234).

**Table 4:** Clinicopathological and prognostic difference among 21 rheumatoid arthritis (RA) patients with other iatrogenic immunodeficiency-associated (OIIA) T- and NK-cell lymphoproliferative disorders (TNK-LPDs), 39 with OIIA B-LPDs and 22 with non-OIIA B-LPDs

	OIIA TNK-LPDs	OIIA B-LPDs	non-OIIA B-LPDs	OIIA TNK- vs. B-LPDs *(p*-value*)*	OIIA TNK- vs. non-OIIA B-LPDs *(p*-value*)*
No. of patients	21	39	22	nt	nt
Age, years, median	64	67	68	0.438	0.108
Sex, female: male	15:6	32:07:00	19:3	0.532	0.281
Nodal: extranodal	13:8	14:25	8:14	0.097	0.131
RA duration, median (months)	72	166	177	0.008	0.0007
MTX therapy	21 (100%)	38 (97%)	0	1	nt
MTX duration, median (months)	53	67	0	0.194	nt
Biologic drugs	4 (19%)	8 (21%)	0	0.84	0.042
LDH, median (U/L)	584	417.2	673	0.105	0.91
LDH ≥ 300 U/L	13 (62%)	13/33 (39%)	14 (64%)	0.182	0.843
sIL2R, median (U/ml)	7588	1623	5731	0.072	0.289
sIL2R ≥ 2000 U/ml	10/19 (53%)	6/28 (21%)	11/16 (69%)	0.025	0.533
Stages III and IV	16 (76%)	17 (44%)	12 (57%)	0.032	0.52
Regression of LPDs^a^	12 (57%)	15 (38%)	0	0.265	nt
CD30 ≥ 30%	7 (33%)	22 (56%)	3 (13%)	0.151	0.162
p53 ≥ 50%	8 (38%)	17/29 (59%)	9/14 (64%)	0.25	0.176
CMYC ≥ 50%	5 (24%)	7/29 (24%)	6/13 (46%)	0.757	0.262
EBER+ atypical lymphocytes ≥ 50%	5 (24%)	21 (54%)	1 (4%)	0.031	0.185
EBV latency infection patterns I/II/III	1/4/0	0/17/4	0/1/0	0.002	0.178
EBER+ atypical and reactive lymphocytes	15 (71%)	27 (69%)	3 (14%)	0.91	0.0002
Chemotherapies	8 (38%)	20 (51%)	20 (91%)	0.743	0.0004
Five-year survival	57%	68%	40%	0.083	0.234

*EBER* EBV-encoded RNA, *LDH* lactate dehydrogenase, *MTX* methotrexate, *sIL2R* soluble interleukin 2 receptor

^a^regression of LPDs by withdrawal of MTX and biologic drugs. *p*-values were analysed using the Fisher's and Wilcoxon rank sum tests, and 5-year survival was using the log-rank and generalized Wilcoxon tests. nt, not test

## Discussion

Seven of our examined 21 RA patients (33%) had OIIA CD4+ TFH+ T-LPDs, and 9 (43%) had complicated CD8+ T-LPDs. EBER+ atypical lymphocytes, scattered EBER+ small lymphocytes, and some large B lymphocytes were detected in six of seven patients with CD4+ TFH+ T-LPDs and in five with CD8+ T-LPDs. A previous study demonstrated that 19 of 28 RA patients with OIIA T-LPDs (68%) showed histological features of CD4+ TFH+ AITL with scattered EBER+ lymphocytes in the background [8]. In RA patients, increased TFH cells and decreased regulatory T cells in the peripheral blood were significantly correlated with the disease activity of RA (*p* < 0.05), and TFH cells were frequently found in the germinal centres of the involved synovial tissue [20]. In RA patients treated with MTX and biologic drugs, EBV-specific effector memory CD8+ T cells were positively correlated with increased EBV viral load in the peripheral blood [21]. It was suggested that abnormal proliferation of CD4+ TFH cells and CD8+ T cells was due to persistent RA activity and EBV infection found in the immunosuppressive states of RA patients treated with MTX and biologic drugs.

In the current study, five of 21 RA patients with OIIA TNK-LPDs (24%) showed a lethal progressive clinical course within 13 months, having features of TFH+ PTCL, EBV+ CD4+ CD30+ large-cell PTCL-NOS, EBV− CD8+ sALCL, and sEBV+ CD8+ CD30+/− TCLs. Four reported RA patients treated with MTX or MTX and Janus kinase inhibitors suffered from sALCL and EBV+ CD30+ large-cell PTCL, and the three ALK− patients died of the disease within months, while the remaining patient with ALK+ sALCL is alive at 24 months [7, 11, 12, 22]. Parakkal et al. [13] reported that 3 RA patients treated with MTX and TNF inhibitors for 1.2 to 5 years as well as 22 patients with IBD receiving TNF inhibitors and azathioprine had hepatosplenic TCL in the FDA database, and 2 RA patients died of the disease. The current and previous studies confirmed that RA patients treated with MTX and TNF inhibitors occasionally had complicated T-LPDs with a lethal progressive clinical course.

Our two examined RA patients (Case Nos 15, 16) treated with MTX-complicated CD8+ T-LGL showed indolent clinicopathological features without chemotherapy [1]. Schwaneck et al. [10] reported that RA patients with mainly CD8+ and occasionally CD4+ T-LGLs mostly showed an indolent clinical course following rituximab (anti-CD20) and anti-IL6 receptor Ab treatment in addition to continuous MTX therapy and/or TNF inhibitors. They suggested that RA-associated T-LGL occurred independently of MTX therapy for RA, and probably due to TNF inhibitors, because of decreasing circulating T-LGLs after cessation of TNF inhibitors. Andrade et al. [23] reported that one RA patient treated with MTX and TNF inhibitor (etanercept) showed CD4+ CD8+ TCL with HLH and scattered EBV+ cells and received prompt chemotherapy, remaining alive at 24 months. The current study demonstrated that two RA patients (Case Nos. 13, 14) treated with MTX-complicated sEBV+ CD8+ TCL died within 4 months, and another patient treated with MTX and TNF inhibitor (infliximab) (Case No. 18) showed CD8+ T-LPD with features of EBV− HLH. Even in post-transplant LPDs, only a few patients showed sEBV+ CD8+ TCL with a lethal progressive clinical course and CD8+ T-LPDs with features of HLH [24–26]. These findings demonstrated that T-LGL with an indolent clinical course was occasionally found, and rare types of lethal and curable EBV+/− CD8+ TCL or T-LPDs with HLH were also detected in RA patients treated with MTX and TNF inhibitors.

The current study demonstrated that clonal peaks of *TCRγ* were detected in 9 of 17 RA patients with OIIA TNK-LPDs (53%). Only one patient among our examined seven with TFH+ TCL and T-LPDs (14%) showed the *RHOA* p.G17V mutation. Twenty-one of 47 RA patients with OIIA B-LPDs (45%) showed rearrangements of the immunoglobulin heavy chain (*IGH*) gene in FFPE specimens by PCR [27], and the clonality was associated with poor recurrence-free survival (*p* = 0.05), but not with OS. It was suggested that frequent spontaneous regression of TNK-LPDs as well as B-LPDs by MTX withdrawal in RA patients occurred due to abnormal but not overt neoplastic proliferation of lymphocytes. However, seven RA patients with MTX-associated TNK-LPDs (33%) died of the disease. Notably, ≥ 500 U/L LDH was a significant poor prognostic factor of PFS and OS in RA patients with OIIA TNK-LPDs (*p* < 0.05). Careful follow-up of serum LDH as well as sIL2R is necessary to detect the progression of OIIA TNK-LPDs in the early-phase of the disease. Histologically, p53 expression in 57 patients with TNKCL was positively correlated with *TP53* mutation variant allele frequency, and p53 was a significant poor prognostic factor (*p* = 0.009) [28]. CMYC expression in patients with aggressive-type adult T-cell leukaemia/lymphoma (ATLL) was significantly higher than that in patients with smouldering and chronic types (*p* < 0.01) [29]. Because large-sized, CD30+, p53+, CMYC+, and EBER+ atypical lymphocytes were identified as poor prognostic factors in RA patients with OIIA TNK-LPDs (*p* < 0.05), timely treatment with chemotherapy and biologic drugs is necessary for TNK-LPD patients with a progressive clinical course having some of the identified prognostic factors.

EBER+ atypical lymphocytes and scattered EBER+ lymphocytes were frequently found in the15 examined patients with OIIA TNK-LPDs (71%), and the findings have been also reported in RA patients with OIIA TNK-LPDs (89%) and B-LPDs (63, 45%), but rarely in RA patients with non-OIIA B-LPDs (8%) [3, 4, 8]. Ejima-Yamada et al. [30] reported that spontaneous regression of LPDs by withdrawal of MTX and biologic drugs was frequently found in 15 of 21 RA patients with OIIA EBV+ B-LPDs (71%), as opposed to none of 17 OIIA EBV− B-LPDs (0%) (*p* < 0.01). Another recent study demonstrated that high EBV real-time PCR value, improvement of lymphocyte counts in the peripheral blood, and patients with EBER+ cells in the involved tissues were significantly associated with the spontaneous regression of LPDs by withdrawal of MTX and biologic drugs in 34 RA patients with LPDs (*p* = 0.003, *p* = 0.036, and *p* = 0.032, respectively) [31]. These findings highly suggest that tumour microenvironments and lymphomagenesis caused by EBV in RA patients occurred in the immunosuppressive states caused by MTX, THF inhibitors, and other biologic drugs.

Bayda et al. [32] demonstrated that strong expression of EBV-produced Bam-HI A rightward transcripts (*BART*s) and early lytic gene, *BNLF2*, were frequently found in 14 AITL patients compared with 21 other types of T-, B- and Hodgkin lymphomas by next-generation sequencing for EBV transcriptomes, and the two genes contributed to immune escape and survival of the infected cells. Furthermore, frequent deletion of *BART* of EBV in peripheral blood mononuclear cells was detected in 10 of 23 patients with TNKCL (44%) and 10 of 14 EBV+ large B-cell lymphoma (71%), but not in 15 with infectious mononucleosis and 32 with post-transplant LPDs [33]. To determine the detailed influence of EBV infection, it is necessary to examine the high expression or deletion of EBV *BART* and *BNLF2* genes in peripheral blood mononuclear cells and tumour tissues between RA patients with OIIA LPDs having regression and no regression of LPDs by withdrawal of MTX and biologic drugs.

This study has some limitations. It was a retrospective study based on small numbers of rare TNK-LPDs in RA patients treated with different types of therapies for LPDs. Hence, it was difficult to confirm the definitive prognostic factors and etiological roles of EBV in TNK-LPDs of RA patients.

## Conclusions

We investigated the clinicopathological characteristics and prognostic factors of 21 RA patients with OIIA TNK-LPDs. Immunohistologically, seven CD4+ TLPD patients (33%) had TFH+ atypical lymphocytes, nine (43%) showed CD8+ T-LPDs, and five (24%) had EBER+ large, atypical lymphocytes. Two patients with TFH+ and EBV+ CD4+ CD30+ PTCL and three with CD8+ ALK− sALCL and sEBV+ CD8+ TCL showed a lethal progressive clinical course within 13 months. Furthermore, ≥ 500 U/L LDH, large, atypical lymphocytes, and CD30, p53, CMYC, and EBER expression in atypical lymphocytes were identified as significant poor prognostic factors for OS (*p* < 0.05). Detection of OIIA TNK-LPDs with poor prognostic factors is required to improve patient outcome. EBER+ atypical and reactive lymphocytes were found in the 15 patients with TNK-LPDs (71%) as well as in 27 of the examined 39 OIIA B-LPDs (69%) but was rare in 22 non-OIIA B-LPDs (14%). EBV infection in the immunosuppressive state resulting from persistent RA and treatment with MTX, TNF inhibitors, and other biologic drugs may play a role in forming the tumour microenvironment and lymphomagenesis of TNK-LPDs.

## Supplementary Information

**Additional file 1: Supplemental Table S1.** Antibodies used in this study.

## Data Availability

The datasets used and/or analysed during the current study are available from the corresponding author on reasonable request.

## Abbreviations

AITL: Angioimmunoblastic T-cell lymphoma
ALCL: Anaplastic large cell lymphoma
ALK: Anaplastic lymphoma kinase
B-LPDs: B lymphoproliferative disorders
BM: Bone marrow
CXCL: C-X-C motif chemokine ligand
CTLA: Cytotoxic T lymphocyte antigen
EBV: Epstein–Barr virus
EBER: EBV-associated encoded RNA
HLH: Hemophagocytic lymphohistiocytosis
ICOS: Inducible T-cell co-stimulator
LDH: Lactate dehydrogenase
LGL: Large granular lymphocytosis/leukaemia
LN: Lymph node
LPDs: Lymphoproliferative disorders
MTX: Methotrexate
NK: Natural killer
NOS: Not otherwise specified
OIIA: Other iatrogenic immunodeficiency-associated
PTCL: Peripheral T-cell lymphoma
RA: Rheumatoid arthritis
sIL-2R: Soluble interleukin-2 receptor
TCL: T-cell lymphoma
TCR: T-cell receptor
TFH: T follicular helper
TNF: Tumour necrosis factor
