# Supplementary material for: Clinicopathological findings, prognosis, and Epstein–Barr virus infection in rheumatoid arthritis patients with other iatrogenic immunodeficiency-associated T- and NK-cell lymphoproliferative disorders
Source: BMC Cancer. 2022 Dec 22;22:1342. doi: 10.1186/s12885-022-10358-0 (PMC9773610; doi:10.1186/s12885-022-10358-0)
Supplement: Supplementary file 1 [file 12885_2022_10358_MOESM1_ESM.docx]

**Table S1.** Primary antibodies used in this study		
Antibodies	Clone	Source
CD3	PS1	Leica, Newcastle, UK
CD4	4B12	Leica, Newcastle, UK
CD8	C81/44B	Leica, Newcastle, UK
T-cell intracytoplasmic antigen (TIA) 1	2GP	Beckman Coulter, Marseille, France
Programmed cell death (PD)-1	NAT105	Abcam, Cambridge, MA, USA
PD-ligand (PD-L) 1	E1L3N	Cell Signaling, Danvers, MA, USA
inducible T-cell co-stimulator (ICOS)	D1K2T	Cell Signaling, Danvers, MA, USA
BCL6	LN22	Leica, Newcastle, UK
C-X-C motif chemokine ligand (CXCL)13	BLC	R&D, Minneapolis, MN, USA
CD10	56C6	Leica, Newcastle, UK
CD246 (anaplastic lymphoma kinase protein)	ALK1	Dako, Glostrup, Denmark
CD30	BerH2	Dako, Glostrup, Denmark
CD25	4C9	Leica, Newcastle, UK
T cell receptor (TCR)βF1	8A3	Pierce-Endogen, Rockford, IL, USA
CD56	1B6	Leica, Newcastle, UK
p53	DO7	Leica, Newcastle, UK
CMYC	Y69	Abcam, Cambridge, MA, USA
CD20	L26	Nichirei, Tokyo, Japan
Latent membrane protein (LMP) 1	CS1-4	Dako, Glostrup, Denmark
EBV nuclear antigen (EBNA) 2	PE2	Leica, Newcastle, UK
